# Speech Sound Production in Adults with Dyslexia

**DOI:** 10.3390/brainsci16050448

**Published:** 2026-04-23

**Authors:** Sabrina Turker, Natalia Kartushina, Narly Golestani

**Affiliations:** 1 Brain and Language Lab, Department of Behavioral and Cognitive Biology, University of Vienna, 1030 Vienna, Austria; narly.golestani@univie.ac.at; 2 Research Group Cognition & Plasticity, Max Planck Institute for Human Cognitive and Brain Sciences, 04103 Leipzig, Germany; 3 Department of Linguistics and Scandinavian Studies, University of Oslo, NO-0317 Oslo, Norway; natalia.kartushina@iln.uio.no; 4 Institute of Developmental and Educational Psychology, Faculty of Psychology, University of Vienna, 1090 Vienna, Austria; 5 Department of Psychology, Faculty of Psychology and Educational Sciences, University of Geneva, 1205 Geneva, Switzerland

**Keywords:** developmental dyslexia, speech production, auditory processing, speech perception, phonemic category size

## Abstract

**Highlights:**

**What are the main findings?**

- Speech production is largely preserved in adult dyslexia, with no robust group differences in voice onset time or phonemic category size under simple reading conditions.
- Adults with dyslexia showed a tendency toward larger acoustic separation between voiced and voiceless stop categories, suggesting potential subtle differences in phonemic category structure.

**What are the implications of the main findings?**

- Subtle alterations in phonemic category structure may occur in dyslexia despite otherwise typical speech production, highlighting the value of continuous acoustic measures.
- Phonetic production metrics may provide complementary insight into phonological representations in dyslexia, motivating future studies that integrate perception and production measures in larger samples.

**Abstract:**

**Background:** Dyslexia is a reading disorder that is associated with phonological processing and awareness difficulties. However, little is known about phonetic production in dyslexia. Whereas individual differences in speech sound perception were linked to native and foreign speech sound production in typical readers, this remains to be explored in dyslexia. Given the phonetic processing deficits frequently encountered in dyslexia, we aimed to pinpoint potential differences in the acoustic realization of native phonemic production in adults with dyslexia. **Methods:** Ten adults with dyslexia and ten age-matched typical readers produced 24 native-language minimal voiced–voiceless word pairs across three places of articulation (labial, dental, velar) in a reading task. Acoustic analyses addressed phonemic category size, between-category distance, and voice onset time (VOT). Pseudoword reading performance served as an index of phonological decoding ability. **Results:** For category size, we observed a trend-level group-by-type interaction (*p* = 0.059, *η*^2^ = 0.04): both groups showed larger category sizes for voiced than voiceless consonants, but this difference was numerically larger in typical readers. Between-category distance showed a marginal group effect (*p* = 0.089, *η*^2^ = 0.14), with larger differences between categories in dyslexia. VOT showed the expected effect of voicing, but no group differences. **Conclusions:** Our results indicate broadly preserved speech production in dyslexia, alongside subtle differences in category separation and size in dyslexia, marked by considerable inter-individual variability.

## 1. Introduction

In spoken language, phonemes, the smallest units of sound that distinguish meaning, can be realized as subtly different variants known as allophones. For instance, the /t/ in “tennis” (aspirated [t^h^]) differs from the /t/ in “start” (unaspirated [t]) or “butter” (flapped [ɾ] in American English). Yet these variations do not change word meaning and are typically ignored by fluent speakers. Over time, individuals accumulate exemplars of speech sounds (i.e., detailed memory traces of speech) and extract abstract representations from these perceived exemplars (phonemes, syllables, prosodic units) to create phonemic categories [1]. During early language development, infants initially perceive all subtle distinctions in the phonemic speech input, but with exposure to their native language, they gradually learn to disregard allophonic variation and focus only on phonemic contrasts that convey differences in meaning, building language-specific speech categories [2,3]. This so-called perceptual tuning is essential for efficient speech processing and later reading acquisition, where mapping letters to stable phonemic categories is critical [4]. As a consequence, disruptions in speech processing can be detrimental to literacy acquisition and fluent reading, resulting in reading impairments [5].

The links between speech perception and production in typical readers have been thoroughly discussed in the literature, with accumulating evidence for a strong association between the two: they draw on partially overlapping cognitive and neural resources and require simultaneous, fast processing of speech on sensory and motor levels [6,7]. While discussing the various production-perception frameworks [8] goes far beyond the scope of this paper (see summary in [6]), it is important to further discuss suggestions regarding the overlap between speech production and perception when it comes to phonemic categories and distances between categories. Previous research with neurotypical readers suggests that speech perception and production abilities are linked, such that individuals who perceive contrasts more accurately also tend to produce them more consistently [9,10]. In line with this relationship, more consistent (i.e., compact, less variable) native phonetic production has been related to individual differences in the production and perception of native and foreign speech sounds: several studies show that people who produce native speech sounds in a more compact way (i.e., who therefore might have more distinct phonemic categories) are better at discriminating and producing similar contrasts. There are different explanations for these links. More compact (i.e., smaller) phonemic categories could be related to better-defined abstract phonemic representations [10] and to better perception skills in L1. This could, in turn, result in better discrimination between L1 and similar L2s. Alternatively, individuals who produce native speech sounds in a more compact and precise way may have better motor control, which could also help them produce foreign speech sounds more accurately. Interestingly, foreign phonemic categories in speech production, especially their sizes, have been shown to be modulated and made more compact by training [11]. The potential to alter or modulate phonemic categories in both perception and production could be highly relevant for disorders known to present with phonological deficits, such as dyslexia. Critically, if native phonemic categories differ in how they are perceived and represented, such differences may also be reflected in the finer acoustics and structure of produced speech categories, even if more global aspects of speech production appear (i.e., sound) largely intact.

In the present work, we focus on individuals with dyslexia, defined as a specific learning disability that affects reading and spelling [12,13,14]. Dyslexia stems from multiple cognitive and linguistic weaknesses [15,16], including poorly specified representations of speech sounds [17]. In fact, phonological deficits are the most frequently encountered deficit in dyslexia [18,19], making dyslexia a highly interesting case for investigating phonemic categories. For example, a longitudinal study with children at risk for dyslexia recently identified poor phonemic representations as a major risk factor that may lead to dyslexia in conjunction with neurobiological deficits [20].

Several views exist on the origin of phonological deficits in dyslexia. For example, it has been shown that individuals with dyslexia struggle to form consistent sound-letter associations, hindering phonemic awareness (i.e., the awareness that speech is made up of discrete sound units) and decoding skills that are foundational for reading [21,22]. Another view proposes that the underlying deficit is one of lower-level, auditory processing; research has shown that individuals with dyslexia have difficulty processing rapidly changing sounds, whether they be speech or non-speech [23]. The temporal sampling framework further proposes atypical neural entrainment to speech rhythms, especially in the delta/theta bands, in dyslexia [5]. There is evidence that auditory temporal processing deficits affect speech and not non-speech, but deficits might extend to non-speech as well [23,24]. Yet other views suggest that the phonological representations themselves are intact, but that access to them is impaired [25].

While much is known about phonemic production in typical readers, very little is known about speech production in individuals with dyslexia. Of the existing studies, a recent one has argued for difficulties in both phonological production and perception; 3-year-old Dutch children at risk for dyslexia showed differences in speech perception (less consistent categorization of stop consonants and less demarcated phoneme boundaries) and in production (percentage of correct consonants and the phonological mean length of utterance) compared to children without a risk [26]. Another study revealed differences between 9–11-year-old children with and without dyslexia when they had to produce multi-syllabic spoken phrases [27]. However, the main differences were in the speech amplitude envelope only and not in the pitch contour. Not all studies support such production differences: one study failed to observe differences in speech production in school-aged children (grades 2–3) with and without dyslexia, tested via articulation of two-syllable words containing the stop consonant pairs /d/ and /t/ [28]. Beyond childhood, research on speech production in adults with dyslexia is scarce, and existing evidence suggests that overt production deficits, if present, are likely subtle rather than categorical [29].

The present study seeks to further investigate potential differences in speech sound production in diagnosed adults with dyslexia, with a focus on how phonemic category structure is implemented during speech production. Given the above-described research showing an association between more variable native phonetic production and poorer perception of speech sounds in typical readers, we hypothesized that in dyslexia, the phonological deficit comes hand in hand with more variable production of native speech sounds. In that sense, we hypothesized that phonemic categories would be larger (i.e., less compact in size), and this should be linked to poorer phonological skills. To this end, adults with dyslexia and age-matched typical readers produced 24 minimal voiced–voiceless word pairs including labial (/p/ vs. /b/), dental (/t/ vs. /d/), and velar (/k/ vs. /g/) stop consonants in a self-paced word reading task. Plosive stop consonants were selected as stimuli because their reliance on rapid spectrotemporal transitions makes them particularly susceptible to the temporal processing and categorical perception deficits well-documented in dyslexia [23]. Based on prior work linking phonetic perception, production, and phonological representations, we computed voice onset time (VOT) to examine phonemic category size and between-category distance (i.e., the absolute distance in ms between the average VOT for voiced and voiceless phonemic categories, computed separately for each participant and for each place of articulation). We further explored whether individual differences in acoustic measures within the dyslexia group were related to phonological decoding ability, indexed by pseudoword reading performance. To foreshadow our results, we observed largely preserved speech production in adults with dyslexia, alongside subtle and variable differences in phonemic category structure, most notably in between-category distance.

## 2. Materials and Methods

### 2.1. Participants

Twenty native French-speaking adults participated in the study. The experimental group consisted of 10 individuals (6 females) with a formal diagnosis of developmental dyslexia (mean age = 25.8 years, SD = 3.4). All participants with dyslexia had received a clinical diagnosis in Switzerland, based on standardized assessments of reading and spelling, and reported persistent reading difficulties despite adequate educational opportunity. Individuals with a history of speech sound disorder, developmental language disorder, attention-deficit/hyperactivity disorder, or other neurodevelopmental or neurological diagnoses were excluded.

The control group included 10 age-matched typical adult readers (6 females) with no history of language or reading disorder (mean age = 23.8 years, SD = 2.9). Group differences in age were not statistically significant (*p* > 0.1). All participants reported normal or corrected-to-normal vision and no known hearing impairments. Informed consent was obtained from all participants prior to participation, in accordance with the ethical guidelines approved by the Commission cantonale d’éthique of Lausanne, Switzerland.

### 2.2. Stimuli

The stimulus set included 24 minimal word pairs representing eight voicing contrasts across three places of articulation: bilabial (/p/–/b/), alveolar (/t/–/d/), and velar (/k/–/g/) (see Table 1). Twenty-four additional fillers were included to mask the focus on voicing; these were matched for word frequency using corpus-based book frequency norms [30].

### 2.3. Procedure

Participants completed a self-paced word-reading task in which they were instructed to read each word aloud as naturally and clearly as possible. Each of the 48-word pairs (24 minimal pairs + 24 fillers) was presented five times, yielding a total of 240 trials per participant (i.e., 120 trials of minimal pairs). Stimuli were displayed visually on a computer screen, and speech was recorded using a Marantz Pro PMD 620 (Manufacturer: Marantz Professional/D&M Holdings Inc. Manufacturing Site: Shirakawa Audio Works, Shirakawa, Japan. Sourcing City/Country: The product was designed and manufactured in Japan, with technical support offices listed in Itasca, Illinois (USA)).recorder at a 44.1 kHz sampling rate, 16-bit resolution, mono channel, with input gain calibrated to −6 dB.

In addition, participants performed a pseudoword reading task, where the number of correctly read pseudowords and the time needed to read these pseudowords were analyzed. Pseudoword reading performance is commonly used as an index of phonological decoding ability, a core difficulty in dyslexia, and was therefore used as a behavioral measure for exploratory correlational analyses with speech production metrics (see Section 2.4).

### 2.4. Acoustic and Statistical Analysis

Speech data were segmented and analyzed using Praat [31]. For each token in the minimal pairs, the VOT was measured manually. The burst release was marked at the initial sharp increase in waveform amplitude, while the onset of voicing was identified at the beginning of the first clear periodic cycle of vocal fold vibration, following the criteria established by Ladefoged [32].

The three chosen acoustic measures were

1. Phonemic category size: Phonemic category size was defined as the range of VOT values, in milliseconds (ms), within ±1 standard deviation from the individual’s mean VOT for each category (voiced and voiceless). Hence, there were six category size measures (one for each consonant) per subject.
2. Between-category distance: Between-category distance was defined as the absolute VOT difference (also in ms) between the mean values of each voiced-voiceless pair within a contrast, resulting in three between-category distance measures per subject.
3. VOT: For VOT, we used VOT values for each voiced and voiceless consonant in the three respective places of articulation, for a total of 120 VOT values per subject. The onset of the stop burst was identified in the waveform as a sudden increase in amplitude corresponding to the release of the stop closure, and confirmed in the spectrogram by a broadband burst of energy. The onset of voicing was defined as the beginning of the first clear periodic cycle of vocal fold vibration, visible as the emergence of regular vertical striations in the spectrogram and corresponding periodicity in the waveform. VOT was calculated as the temporal interval between burst release and the onset of periodic voicing.

Linear mixed-effects models (LMMs) were used to examine the effects of group (typical vs. dyslexia), consonant type (voiced vs. voiceless), place of articulation (dental, labial, velar), and their interactions on the dependent variables category size, category distance, and VOT. All models were fitted with the lme4 and lmerTest (version 1.1.37) packages and using maximum likelihood estimation in R (version 4.4.2) [33]. Please note that model fit was tested and compared between models. Fixed effects included the main effects of the relevant predictors and their interactions, depending on the model. Random intercepts for participants were included in all models to account for variability between participants, and random intercepts for items were included in the VOT model to account for differences in consonant category production across words. All dependent variables (category size, between-category distance, and VOT) were analyzed on their original scale. Model assumptions were evaluated for all three mixed-effects models (VOT, phonemic category size, and between-category distance). For each model, residual-versus-fitted plots indicated that residuals were symmetrically distributed around zero across the fitted range, with a flat loess trend line, suggesting no violations of linearity or homoscedasticity. Visual inspection of Q-Q plots further confirmed that residuals were approximately normally distributed. Taken together, these diagnostics indicated that the assumptions of the linear mixed-effects framework were adequately met, and no data transformation was required. Since sex did not improve any model fit in the model pruning process, it was not included in the final models. The final LMMs were as follows:

(1) Category size~group × type + (1 | subject).
(2) Category distance~group × place + (1 | subject).
(3) VOT~group × type × place + (type || subject) + (1 | item).

The significance of fixed effects and interaction was tested using Type III ANOVA with Satterthwaite’s approximation for degrees of freedom, with the R function anova(model_X) from the *car* package [34]. Only this output is reported in the results section. For the full model report, see the OSF project page. For all model results, we report effect sizes computed with the R function eta_squared. Note that conventional benchmarks for effect size interpretations are the following: small effect: *η*^2^ = 0.01, moderate effect: *η*^2^ = 0.06, large effect: *η*^2^ = 0.14) [35]. All post hoc pairwise comparisons were conducted using *emmeans* [36], with Tukey HSD adjustment.

Given non-normality in the distribution of variables, exploratory Spearman correlations were computed between averaged speech production measures (category size, between-category distance, VOT voiceless, VOT voiced) and each pseudoword reading measure (accuracy and reading time) within the dyslexia group only. Regarding the correlations, several variables were non-normally distributed, which is why Spearman’s *rho* was used. The *p*-values were adjusted for multiple comparisons using false discovery rate (FDR) correction. Although dyslexia is often conceptualized as lying on a continuum of reading ability rather than as a discrete category [13], we chose to perform within-group associations in the dyslexia group only. This decision was motivated by the limited sample size and by concerns that partialling out group membership in a mixed sample can distort correlation estimates when group differences are meaningful and non-random [37]. Moreover, partial correlations are suggested to be more fragile with such small samples [38]. We report only correlations with an absolute magnitude of at least ρ = 0.30, reflecting at least moderate associations. All correlations are exploratory and did not reach statistical significance after correction for multiple comparisons. Beforehand, we checked performance on pseudoword reading (accuracy, time) between groups. Given violations of normality assumptions, non-parametric Wilcoxon rank-sum tests were used to assess group differences in pseudoword reading performance.

## 3. Results

For phonemic category size, we observed a significant main effect of consonant type (F_(1, 5.88)_ = 15.00, *p* = 0.009, *η*^2^ = 0.72), showing that voiced stops have larger, less compact phonemic categories than voiceless stops. Descriptively, phonemic category sizes were larger for voiced than voiceless stops in both typical readers (83.2 ms vs. 51.6 ms) and participants with dyslexia (76.1 ms vs. 56.7 ms).

**Figure 1:**
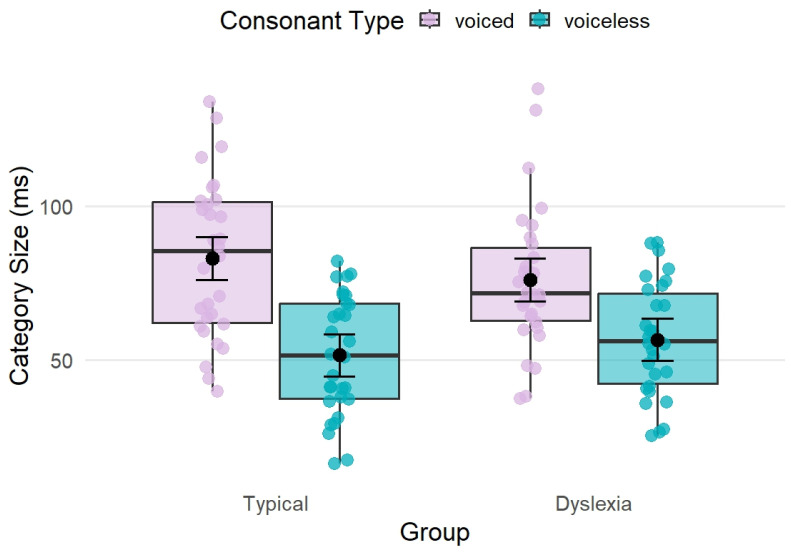
Visualization of category sizes for the two types of consonants (voiced [in lilac] vs. voiceless [in cyan]) for both groups (typical vs. dyslexic). Category size is shown in ms.

We found no main effect of group (F_(1, 19.73)_ = 0.03, *p* = 0.865, *η*^2^ < 0.001), but the group by type interaction reached marginal significance (F_(1, 94.67)_ = 3.67, *p* = 0.059, *η*^2^ = 0.04). To further explore this marginal interaction, we performed post hoc tests. These revealed a robust difference between voiced and voiceless categories in typical readers (*t* = 3.737, *p* = 0.003), and a smaller but still significant difference in the dyslexia group (*t* = 2.294, *p* = 0.041). This suggests that the voiced-voiceless contrast in category size was numerically larger in typical readers than in participants with dyslexia. The results are summarized in Table 2 and Table 3 and Figure 1.

**Figure 2:**
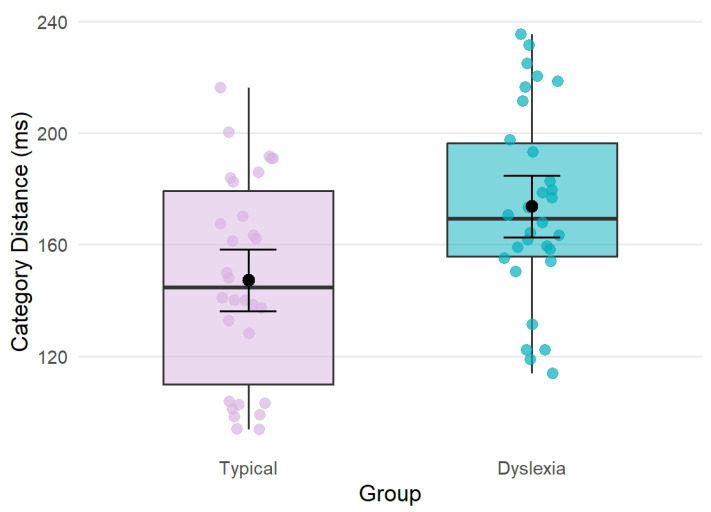
Between-category distance (i.e., the difference in mean VOT between voiced–voiceless pairs) in typical readers (lilac) and individuals with dyslexia (cyan). Category distance is shown in ms. Individual dots represent individual subject-level category distance values for each place of articulation.

**Figure 3:**
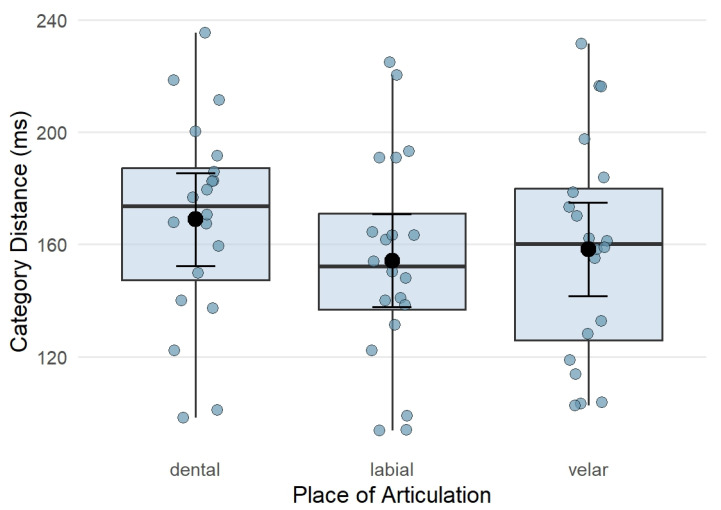
Between-category distance (in VOT) for each place of articulation (dental, labial, velar). Category distance is shown in ms. Individual dots represent individual category distances per subject.

The analysis examining the effects of group (typical vs. dyslexia) and place of articulation (labial, dental, velar) on between-category distance (i.e., the difference in mean VOT between voiced–voiceless pairs within each contrast) revealed a marginal main effect of group (F_(1, 20)_ = 3.20, *p* = 0.089) of large effect size (*η*^2^ = 0.14), with adults with dyslexia tending to show larger distances between voiced and voiceless categories than typical readers. There was also a significant main effect of place (F_(2, 40)_ = 9.97, *p* < 0.001, *η*^2^ = 0.33), indicating that between-category distance (in VOT) differed across places of articulation. Post hoc pairwise comparisons for the main effect of place showed that dental contrasts had larger between-category distances than labial contrasts (*t* = 4.10, *p* < 0.001), and that dental contrasts also had larger between-category distances than velar contrasts (*t* = 2.97, *p* = 0.013). Labial and velar contrasts did not differ significantly (*t* = −1.14, *p* = 0.497). The group-by-place interaction was not significant (F_(2, 40)_ = 0.27, *p* = 0.766, *η*^2^ = 0.01). Model results and post hoc comparisons are presented in Table 4 and Table 5, Figure 2 and Figure 3.

**Figure 4:**
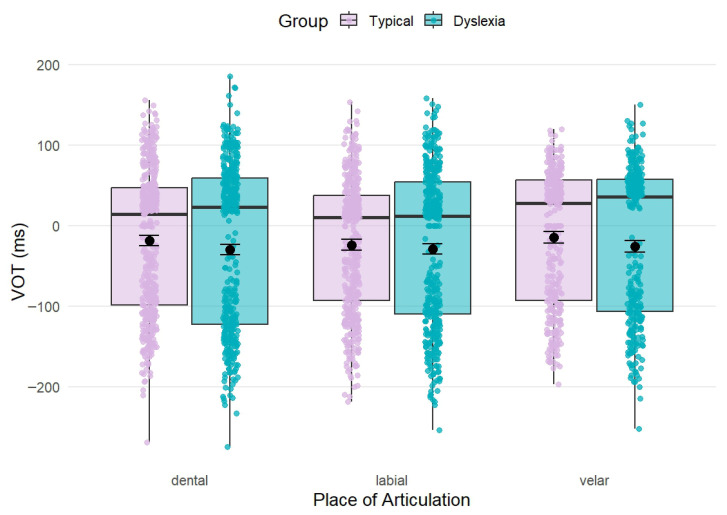
Illustration of variability in VOT, shown for both groups (typical readers: lilac, dyslexic group: cyan) and each place of articulation. Individual dots represent individual consonant productions across the whole sample.

The model examining the effects of group, type and place of articulation on VOT revealed a highly significant main effect of type (*F*_(1, 42.40)_ = 291.79, *p* < 0.001, *η*^2^ = 0.87), with voiced stops showing shorter VOTs than voiceless stops. Neither the main effect of group (*F*_(1, 19.73)_ = 2.75, *p* = 0.113, *η*^2^ = 0.12), nor the main effect of place (*F*_(2, 43.59)_ = 0.35, *p* = 0.709, *η*^2^ = 0.02) reached significance. However, we observed a marginal interaction between group and place (*F*_(2, 2351.32)_ = 2.77, *p* = 0.063, *η*^2^ < 0.001), but the associated effect size was negligible (*η*^2^ < 0.001). None of the other interactions were significant (Table 6). A visual summary of VOT distributions is presented in Figure 4.

Exploratory post hoc comparisons were conducted to examine potential group differences in VOT within each place of articulation. For dental stops, typical readers produced numerically longer VOTs than participants with dyslexia (11.52 ms), a difference that approached but did not reach significance (t_(25.13)_ = 1.91, *p* = 0.068). For labial stops, the group difference was smaller and clearly non-significant (5.28 ms; t_(25.02)_ = 0.88, *p* = 0.390). For velar stops, typical readers again showed numerically longer VOTs than the dyslexia group (10.92 ms), but this difference also remained non-significant (t_(27.34)_ = 1.77, *p* = 0.087). Taken together, these results indicate that VOT production does not differ systematically between groups as a function of place of articulation.

Regarding phonological decoding performance, as expected, participants with dyslexia showed overall lower pseudoword reading accuracy (*M* = 17.00, *SD* = 2.60) and slower pseudoword reading times (*M* = 35.67 s, *SD* = 11.79) compared to typical readers (accuracy: *M* = 19.20, *SD* = 1.03; time: *M* = 18.10 s, *SD* = 4.25) (accuracy: *W* = 15, *p* = 0.01; time: *W* = 88, *p* < 0.001).

Exploratory Spearman correlations were conducted within the dyslexia group to examine whether individual differences in speech production were associated with pseudoword reading performance. Across speech-production measures, several moderate numerical associations emerged, although none reached statistical significance (all ps > 0.10). The following reported correlations are descriptive in nature and should be interpreted cautiously, given the small sample size and their exploratory character. Category size was moderately negatively associated with pseudoword reading accuracy (ρ = −0.52), suggesting that better pseudoword reading might go hand in hand with smaller phonemic categories. In addition, category distance was moderately negatively related to pseudoword reading accuracy (ρ = −0.40), suggesting that greater acoustic separation between voiced and voiceless categories was associated with poorer pseudoword reading. Mean VOT for voiced consonants showed a moderate positive association with pseudoword reading accuracy (ρ = 0.49), i.e., higher VOTs could be linked to better performance. Mean VOT for voiceless consonants was moderately negatively associated with accuracy (ρ = −0.38) and positively related to pseudoword reading time (ρ = 0.49), indicating that participants with higher voiceless VOTs tended to read pseudowords less accurately.

## 4. Discussion

The present study examined whether adults with dyslexia show differences in the production of voicing contrasts, focusing on phonemic category size, between-category distance, and VOT. Overall, the results provide limited but informative evidence for subtle differences in speech sound production in dyslexia. While most effects were weak or non-significant, several patterns emerged that may guide future research using larger samples.

Regarding differences in phonemic category size, we observed a robust effect of voicing, i.e., voiced stop categories were consistently less compact (i.e., larger) than voiceless categories across both groups. This pattern is somewhat surprising given that voiceless stops typically exhibit greater VOT variability due to aspiration-related timing differences, whereas voiced stops tend to show shorter and more stable VOT values [39]. Regarding group differences, the main effect of group on category size was not significant, and the group-by-type interaction only reached marginal significance. Post hoc tests indicated that both typical readers and participants with dyslexia showed significantly larger category sizes for voiced relative to voiceless consonants, although this difference was numerically bigger in typical readers than in adults with dyslexia. Given that the interaction did not reach conventional significance thresholds, these group-related differences should be regarded as exploratory rather than conclusive.

The analysis of between-category distance revealed a significant effect of place of articulation, with dental contrasts showing greater separation between voiced and voiceless categories than velar or labial contrasts. The main effect of group approached significance, with adults with dyslexia tending to show larger between-category distances (i.e., larger acoustic separation between categories) than typical readers. Although this effect did not reach conventional significance thresholds and was not modulated by place of articulation, its direction is noteworthy. One possible interpretation is that adults with dyslexia may produce more strongly separated voicing contrasts. This could point towards an exaggerated differentiation of stop consonants during the simple minimal pair reading task in our dyslexic sample, who were largely university students and therefore likely well-compensated dyslexics. Alternatively, and non-exclusively, this pattern may also be interpreted within the framework of the allophonic theory of dyslexia. Based on earlier research [10], this could also suggest that well-compensated individuals with dyslexia use larger distances to guide or enhance the perceptual distinction of speech sounds in their production. However, given the modest effect size, the substantial overlap between groups and the limited statistical power of the present study, these interpretations remain tentative.

As expected, VOT showed a very large and significant main effect of voicing. In contrast, there was no robust evidence for group differences in VOT production. One interaction (group by place) approached significance, but the associated effect size was negligible, making it unlikely to reflect a meaningful phonetic difference. Post hoc comparisons revealed no statistically significant group differences within individual places of articulation.

Correlational analyses within the dyslexia group revealed no significant associations between acoustic measures and pseudoword reading performance. Although several moderate numerical associations (ρ ≈ 0.3–0.5) emerged, none reached statistical significance and should therefore be interpreted with much caution. Most notably, category size was moderately negatively linked to phonological decoding performance, with smaller category sizes tending to be linked to higher accuracy. Likewise, smaller between-category distances were numerically related to better pseudoword reading performance. Although these relationships were exploratory and non-significant, their direction is qualitatively consistent with previous findings in neurotypical adult readers showing that people who produce native speech sounds more variably have more difficulty in hearing and producing novel, foreign speech sounds [11,40]. Nevertheless, within the present sample, individual differences in speech production were not robustly related to variability in phonological decoding performance, indexed via a pseudoword reading task. Nonetheless, we suggest that this line of research warrants further work at the intersection of phonetic production, phonetic perception, reading and reading disorder in larger samples.

As mentioned above, the present findings can be considered in light of the allophonic theory of dyslexia, which proposes that individuals with dyslexia rely on overly fine-grained phonetic, rather than abstract phonemic, representations (e.g., [18,23]). While our data do not provide strong confirmatory evidence for this theory, they are compatible with the possibility that subtle traces of finer-grained acoustic-phonetic (i.e., allophonic) encoding may be present in speech production in adult dyslexia under certain conditions. Specifically, adults with dyslexia tended to show numerically larger acoustic distances between voiced and voiceless stop categories than typical readers, which may reflect a stronger reliance on phonetic detail when marking phonological contrasts. They also showed qualitatively more compact (i.e., smaller) category sizes for the voiced consonants than the controls. At the same time, neither phonemic category size nor VOT revealed robust group differences, suggesting that speech production in adult dyslexia is largely preserved during simple reading.

Taken together, the present results argue against broad or global impairments in speech production in adult dyslexia. Instead, they point to a nuanced pattern in which subtle alterations in phonemic category structure may coexist with largely intact speech production. The substantial overlap between groups across all measures further suggests that any production-related differences are likely small and variable across individuals. The present findings highlight the importance of examining continuous acoustic-phonetic measures, and underscore the need for future studies with larger samples, more demanding production tasks, and combined perception–production paradigms to more precisely characterize phonological representations in dyslexia.

## 5. Limitations and Future Directions

Several limitations should be considered when interpreting these findings. First and foremost, the sample size was small, which substantially limits statistical power. Although the sample size is comparable to previous phonetic studies in dyslexia, especially in adult populations, the present results should be interpreted with caution and cannot be taken as definitive evidence for or against production differences in dyslexia. Second, the use of a self-paced reading task likely reduced articulatory demands and may not have tapped into processes that become more fragile under time pressure or increased cognitive load. As a result, the task may have underestimated potential production differences that could emerge under more demanding speaking situations, particularly those requiring rapid planning, online monitoring, or increased motor coordination. Third, the phonemic contrasts examined here represent only a subset of the phonological system. While stop consonant voicing contrasts provide a well-controlled and theoretically relevant test case, other contrasts (e.g., fricatives, vowel duration, stress, intonation, or prosodic timing) may reveal different or stronger group differences and should be examined in future work. A fourth limitation concerns the interpretation of group differences in a case–control design. Dyslexia is a heterogeneous condition with substantial individual variability, and the large overlap between groups observed across all measures suggests that any production-related differences are subtle and not categorical. Consequently, the present findings do not support the notion of a uniform speech production deficit in dyslexia but rather point to small shifts in central tendency that may characterize only a subset of individuals. Finally, the correlational analyses linking speech production measures to pseudoword reading performance were exploratory and underpowered. Although several moderate numerical associations emerged, none reached statistical significance, and these patterns should therefore be treated as hypothesis-generating rather than confirmatory.

Future research should consider larger samples, more demanding and more natural production tasks (e.g., rapid naming, nonword repetition, sentence elicitation, narration), and multimodal approaches that integrate perception and production. Longitudinal designs and continuous, dimensional approaches to reading ability may be particularly informative, given increasing evidence that dyslexia lies on a continuum rather than constituting a discrete category. Importantly, more sensitive modeling of phonemic distributions—including probabilistic representations and machine-learning-based clustering—may better capture subtle differences that are not detectable via traditional measures. Such approaches may help clarify whether fine-grained phonetic variability reflects compensatory strategies, residual traces of atypical phonological development, or broader individual differences unrelated to reading outcomes.

## 6. Conclusions

Across measures, the overall pattern of findings suggests that speech production in adult dyslexia is largely preserved, particularly for basic temporal parameters such as VOT for stop consonants. At the same time, we observed small and subtle group-related tendencies in the structure of phonemic categories, which should be interpreted cautiously given the limited sample size and substantial overlap between groups.

Firstly, both groups showed reliably smaller category sizes for voiceless than for voiced consonants, reflecting well-established differences in the acoustic stability of these categories. This voicing-related difference in category size was numerically larger in typical readers than in individuals with dyslexia, but the corresponding interaction did not reach conventional significance and should therefore be regarded as exploratory.

Secondly, a marginal group effect showed that dyslexic participants tended to produce greater separation between voiced and voiceless categories. While this pattern is compatible with the notion of enhanced acoustic differentiation in some individuals with dyslexia, it does not support a categorical or uniform production deficit. Rather, it suggests small shifts in central tendency that may characterize only a subset of individuals with dyslexia. These tendencies could reflect compensatory production strategies in well-adapted adult readers with dyslexia, or, non-exclusively, align with predictions from the allophonic theory of dyslexia, which posits heightened sensitivity to fine-grained phonetic distinctions. Importantly, the present data do not allow causal conclusions regarding the role of phonetic category structure in the development or persistence of dyslexia.

Exploratory correlational analyses within the dyslexia group further indicated moderate numerical associations between acoustic measures and pseudoword reading performance, although none reached statistical significance. These findings should therefore be interpreted as hypothesis-generating rather than confirmatory. Nonetheless, the observed directions of association suggest that greater variability in speech production and increased acoustic separation between phonemic categories may co-occur with less efficient phonological decoding in some individuals.

In sum, these results point to a nuanced picture: adults with dyslexia do not exhibit broad impairments in speech production, but they may show subtle alterations in phonemic category structure that relate to individual differences in reading ability. Crucially, the large overlap between groups underscores that such production-related differences are neither necessary nor sufficient to explain dyslexia at the individual level. These findings underscore the potential value of examining continuous phonetic measures and phonetic production—not only categorical perception—when characterizing phonological representations in dyslexia. Future work should build on these preliminary observations using larger samples, more demanding production tasks, and integrated perception–production paradigms to clarify whether and how fine-grained phonetic representations contribute to reading difficulties within a multifactorial framework of dyslexia.

## Figures and Tables

**Table 1:** Minimal word pairs across three places of articulation.

	Labial		Dental		Velar	
Pair	Word 1	Word 2	Word 1	Word 2	Word 1	Word 2
1	pain	bain	tard	dard	camp	gant
2	pas	bas	thé	dé	car	gare
3	poire	boire	tire	dire	classe	glace
4	poisson	boisson	toit	doigt	con	gong
5	pont	bon	ton	don	court	gour
6	poule	boule	touche	douche	cru	grue
7	pull	bulle	tout	doux	cri	gris
8	paix	baie	tu	du	quai	gai

**Table 2:** Linear mixed model results with category size as dependent variable, including the fixed effects of group (typical vs. dyslexia), type (voiceless vs. voiced) and their interaction.

	numdf	dendf	f	*p*	*η* ^2^
Group	1	19.731	0.0297	0.865	<0.001
Type	1	5.875	15	0.009 **	0.72
Group/Type	1	94.668	3.669	0.059 +	0.04

** *p* < 0.01, + *p* = 0.059.

**Table 3:** Post hoc tests disentangling the group-by-type interaction.

	Contrast	est.	SE	df	t	*p*
typical	voiced vs. voiceless	31.6	8.46	12.1	3.737	0.003 **
dyslexia	voiced vs. voiceless	19.4	8.46	12.1	2.294	0.041 *

** *p* < 0.01, * *p* < 0.05.

**Table 4:** Linear mixed model with between-category distance as the dependent variable, including the fixed effects of group (typical vs. dyslexia), place (labial vs. dental vs. velar), and their interaction.

	numDF	denDF	F	*p*	*η* ^2^
Group	1	20	3.199	0.089 +	0.14
Place	2	40	9.970	<0.001 ***	0.33
Group/Place	2	40	0.269	0.766	0.01

*** *p* < 0.001, + *p* = 0.089.

**Table 5:** Post hoc tests for the main effect of place of articulation (dental, velar, labial) on between-category distance.

Contrast	est.	SE	df	t	*p*
dental vs. labial	14.63	3.57	44.4	4.102	<0.001 ***
dental vs. velar	10.58	3.57	44.4	2.966	0.013 *
labial vs. velar	−4.05	3.57	44.4	−1.137	0.497

*** *p* < 0.001, * *p* < 0.05.

**Table 6:** The effects of group, type and place, as well as their interactions, on VOTs.

	numDF	denDF	F	*p*	*η* ^2^
Group	1	19.73	2.746	0.113	0.12
Type	1	42.40	291.792	<0.001 ***	0.87
Place	2	43.59	0.345	0.709	0.02
Group/Type	1	19.90	2.961	0.101	0.13
Group/Place	2	2351.32	2.767	0.063 +	<0.001
Place/Type	2	43.59	0.673	0.515	0.03
Group/Type/Place	2	2351.34	0.167	0.847	<0.001

*** *p* < 0.001, + *p* = 0.063.

## Data Availability

The original data and code presented and used in the study are openly available on the OSF project page: https://osf.io/4a6zh/ (accessed on 20 April 2026).

## Abbreviations

The following abbreviations are used in this manuscript:

LMM	Linear Mixed Model
VOT	Voice Onset Time
